# Integrating “omics” Technologies to Conceptualize Dynamic Antimicrobial Peptide Responses

**DOI:** 10.3389/fimmu.2012.00284

**Published:** 2012-09-17

**Authors:** Jennifer K. Plichta, Vanessa Nienhouse, Katherine A. Radek

**Affiliations:** ^1^Department of Surgery, Burn and Shock Trauma Institute, Loyola University Health SystemsMaywood, IL, USA

## Introduction

Rarely in human disease can a single host factor be identified as the primary causal factor of a disease, syndrome, or disorder. Rather, the clinical manifestations that culminate in a symptomatic state are usually triggered by a perturbation of multiple factors derived from several distinct, but integrated, domains of cellular regulation. Host immune responses, such as antimicrobial peptide (AMP) production and regulation, are the result of interdependent dynamic interactions between host cells, extracellular milieu, and the external environment. In order to truly elucidate disease pathogenesis, it is imperative to consider the entire system at play from a global perspective, which is the promise of “omics” technology. The term “omics” was originally coined as a discipline in molecular biology that examines global sets of biological molecules (Micheel et al., 2012), which consequently stimulated an “Omics Revolution” throughout the scientific community. Genomics emerged following the sequencing of the human genome in the 1990s and early 2000s. This subsequently led to proteomics, which includes the entire complement of proteins and their related structure, modifications, and function. Pauling et al. (1971) proposed that estimating the abundance of metabolites in biological fluids may indicate the functional status of a specific biological system. Consequently, metabolomics has recently surfaced as an approach to comprehensively identify and quantify low molecular weight exogenous and endogenous metabolites and compounds in a biological system using high-throughput methods. Using 16S rRNA gene sequencing tools originally developed in the field of environmental microbiology, we can now generate a site-specific microbiome profile for any given patient. The Human Microbiome Project and numerous other projects aim to discern relationships between human-associated microbes and health and disease (Zoetendal et al., 2006). Such research has generated massive and complex data sets, which requires further advancement and refinement of bioinformatics and biostatistical applications. Often, the data is used to produce computational models to distinguish specific characteristics of a given population. Due to the unavoidable complexities and required statistical sophistication, few medical fields have developed clinically useful applications for the resulting data. Thus, AMPs are an ideal set of biological molecules to assess, since there biological role spans numerous areas of clinical research.

Due to their diverse microbicidal and immunomodulatory functions (Steinstraesser et al., 2011), AMPs comprise a major aspect of the host innate immune response (Nakatsuji and Gallo, 2012). Alterations in AMP production and/or localization are associated with several cutaneous dermatoses. Patients diagnosed with atopic dermatitis and psoriasis ultimately develop epidermal barrier defects and microbial susceptibility, or a hyperproliferative/inflammatory epidermis, respectively (Schauber and Gallo, 2008; Schroder, 2011). The contributions of AMPs to tissue integrity and epithelial defense was also established in the brain (Schluesener et al., 2012; Williams et al., 2012), urinary tract (Saemann et al., 2007; Zasloff, 2007; Ali et al., 2009), gastrointestinal tract (Lisitsyn et al., 2012), and certain malignancies (Kesting et al., 2012; Scola et al., 2012). However, altered AMP regulation does not occur exclusively, but instead embodies dynamic transformations that emerge as a disturbed system. The integration of several different “omics” techniques may provide a more complete understanding of the diverse factors contributing to a specific disease state, responses to pharmacologic agents, or novel alternatives to current treatment modalities.

## AMPs as a Disease Marker

The appeal of “omics” research lies in the possibility of identifying an AMP profile that distinguishes one particular patient in a clinically useful manner to categorize them as high- or low-risk for developing a post-operative infection (e.g., pneumonia). Several studies have demonstrated the importance of cathelicidin (Braff et al., 2007; Kovach et al., 2012), beta-defensins (Chong et al., 2008; Scharf et al., 2012), and several other AMPs in regulating epithelial immunity (Cole and Waring, 2002; Tecle et al., 2010). Consequently, it is feasible that integrating these variables simultaneously may reveal unexplored or intriguing connections, and likely identify invaluable correlations that are clinically significant.

Antimicrobial peptides have been extensively evaluated for their role in inflammation (Lai and Gallo, 2009), while little research has investigated their potential role in rejection following organ transplantation. Although these patients are typically immunosuppressed, abnormal alterations in AMPs following transplantation may contribute to or serve as a marker of inflammation and rejection. A diagnostic tool to yield a molecular AMP profile of a transplant patient could serve as a prognostic indicator of organ failure. Nuclear magnetic resonance (NMR) based metabolomic technologies have also attempted to identify other urine biomarkers as an indicator of chronic renal failure and renal transplant function (Bell et al., 1991; Foxall et al., 1993). Similar technologies could be employed to identify how AMPs may correlate with clinical outcomes in transplant patients. In comparison, trauma and burn patients exhibit profound defects in immune regulation following injury, including perturbations in AMPs (Steinstraesser et al., 2004; Bhat and Milner, 2007). A diagnostic AMP profile may again provide invaluable data to predict healing, immune integrity, or graft survival. The clinical utility of such targeted profiles could undoubtedly be applied to numerous disease states involving infection and/or inflammation, to serve as markers of prognosis.

Currently, NMR and Mass Spectrometry are two major platforms by which metabolomic analyses are evaluated via bioinformatic tools. Several skin AMPs were recently implicated in tumorigenesis of cutaneous squamous cell carcinoma (Scola et al., 2012). Significant metabolic alterations usually ensue as normal cells are transformed into a malignant phenotype. Using a limited rationale, AMPs may simply reflect alterations in the local environment from the presence of the malignancy, or may simply be irrelevant to the malignancy. A more sophisticated rationale suggests that alterations in AMPs may serve as a biomarker for disease severity and/or progression, and denote a significant underlying process that contributes to a malignancy. Interestingly, the wound repair process and cancer progression are both associated with alterations in the inflammatory/immune microenvironment. During wound repair, AMPs are released from epithelial and infiltrating immune cells to stimulate re-epithelialization, new vessel formation, and extracellular matrix (ECM) remodeling (Radek and Gallo, 2007). However, the dynamics of cancer progression and tissue repair differ in that wound healing is a self-limiting process, while tumor formation is characterized by a continuous, uncontrolled activation of similar pathways that facilitate tumor growth and metastasis. One key observation is that prominent associations exist between the cytokines, chemokines, and growth factors present in healing wounds and wound fluid, as compared to tumors. In parallel, a striking difference in the temporal regulation of these factors was determined by the combination of several genomic technologies (Pedersen et al., 2003). Furthermore, proteomics and genomic methods are now being employed through a multidisciplinary translational research approach to improve the bioactive components in matrix therapies for non-healing wounds to specifically modulate the temporal and local release of these micromolecules (Sweitzer et al., 2006). Degradomics is emerging in the wound healing field as a new technology that assimilates the current knowledge database of ECM regulation and deciphers the complex interactions between proteases and their respective inhibitors using systems biology as a means to improve wound integrity in chronic wounds (Hermes et al., 2011). Since AMPs are an integral part of wound healing and inflammation, the knowledge gained from utilizing these evolving “omics” technologies may be extrapolated to other dimensions of data analysis that span other disease states which share similar mechanisms of disease progression.

Antimicrobial peptide regulation can clearly modulate and be influenced by the composition of the microbial flora of the human host. Several AMPs are induced in response to both invasive pathogens, as well as commensal strains of bacteria to generate specific down-stream innate or adaptive immune signaling events. For instance, the cutaneous commensal *Staphylococcus epidermidis* induces human β-defensin-2 and -3 via a TLR-2 signaling dependent mechanism (Lai et al., 2010). This interaction is beneficial for both the host and microbe by facilitating the eradication of pathogens on the skin via AMP induction, while simultaneously allowing *S. epidermidis* to further proliferate with fewer competitors for metabolic resources. Further complicating these interactions, microbes have evolved several mechanisms to evade host AMPs via altered cell surface charge, efflux transporters, proteases, or trapping proteins, and direct adaptations of host cellular processes (Nizet, 2006). These dynamic interactions between the host and the resident microbiota can significantly influence the overall homeostatic balance.

The integration of multiple “omics” disciplines is applicable to several tangible clinical situations where infection is a risk factor. For example, identification of patients most at risk for a urinary tract infection (UTI) would improve prophylactic therapies for susceptible patient populations, including burn-injured, surgical, or bedridden individuals. Recent studies deliver a long overdue confirmation that urine is not sterile, which challenges the current dogma (Nelson et al., 2010; Dong et al., 2011; Wolfe et al., 2012). Thus, integration of multiple “omics” technologies, such as 16S rRNA gene sequencing and proteomics, may identify correlations between specific AMPs and distinct genera of bacteria to identify unique patterns that could be employed as a diagnostic tool to predict those individuals who may be at a higher risk for a UTI. Furthermore, the development of a rapid, high-throughput assay that integrates multiple “omics” technologies to correlate AMPs with tissue specific microbiota would be invaluable to clinicians for prediction of UTI or other disease states.

## AMPs as a Therapeutic Target

Aside from patient-centered applications, AMP-related “omics” research is also being utilized in drug discovery, as AMPs have been targeted as a potential alternative to conventional antibiotics by serving as adjuncts and/or replacements to traditional antibiotics, although more research is clearly needed to develop these promising tools (Hirsch et al., 2008; Baltzer and Brown, 2011; Ahmad et al., 2012; Hassan et al., 2012). Infection remains a leading cause of death in the US, with influenza/pneumonia and septicemia both ranking in the top 15 (Murphy et al., 2012). Several publicly available databases have already been established in order to collect relevant information related focused on AMPs (Brahmachary et al., 2004; Wang and Wang, 2004; Wang et al., 2009; Seshadri Sundararajan et al., 2012) in order to construct models based on clustering and analyzing AMP sequences, which allow accurate recognition of specific antimicrobial classes (Fjell et al., 2007). Recently, a bioinformatics strategy using peptide sequences was employed to classify synthetic and endogenous AMPs based on their physiochemical properties to identify active peptides and assess antimicrobial potency (Kumari et al., 2012; Torrent et al., 2012). Despite promising preliminary data, AMPs have yet to prove their full potential in clinical trials.

## Research Limitations

It is evident that less costly and efficient high-throughput technologies are undoubtedly needed to fully explore the under-utilized and under-recognized potential of AMPs for therapeutic applications. Although it is feasible to capture a given patient's molecular profile, the interpretation of those findings remains a challenge. Given the enormous data sets being generated by “omics” research, the development of useful computational models has been limited by several factors. Primarily, analyses of high-dimensional data are prone to overfitting of the models to the study samples, thus yielding inaccurate results in subsequent follow-up studies. Therefore, replication and reproducible verification become imperative when performing such research. Prospective technological advancement merged with more robust bioinformatic tools and greater data analysis capacity will help surmount the existing limitations to allow for the complete integration of micromolecules with systems biology.

## Concluding Remarks

**Figure 1:**
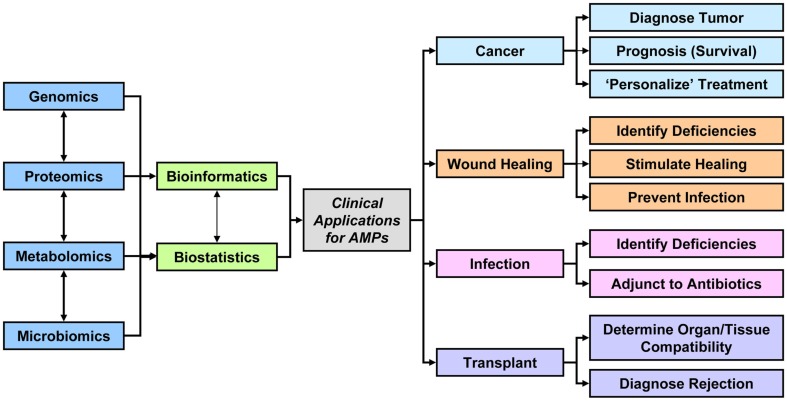
**Data acquired from the combination of genomics, proteomics, metabolomics, microbiomics, and other omics technologies either directly investigating AMPs or examining related parameters may be integrated to design better diagnostic tools, therapeutic options, or prognostic indicators for patients with cancer, wounds, infections, or transplant**.

While the “Omics Revolution” continues to rapidly expand and mature, it is apparent that its maximal applicability and utility remain to be fully elucidated. “Omics” research, either directly examining AMPs or researching AMPs in the context of related factors such as the metabalome or the microbiome, could potentially identify significant and crucial relationships between various molecular signatures and human disease (Figure 1). These enormous data sets may serve as the foundation for the evolution of computational models that could predict disease, complications, or even prognosis based on a specific AMP profile. Furthermore, modified AMPs have the potential to replace current antibiotic therapies, as drug discovery begins incorporating the latest technology into their pharmaceutical development pipelines. Ultimately, the new approach to personalize healthcare can foster novel applications to refine the characterization of a disease phenotype, identify predictive biomarkers, determine the efficacy of various therapies, or determine the susceptibility to drug toxicity for each individual patient.
